# Insecticidal and Cholinesterase Activity of Dichloromethane Extracts of *Tithonia diversifolia* on *Atta cephalotes* Worker Ants (Formicidae: Myrmicinae)

**DOI:** 10.3390/insects11030180

**Published:** 2020-03-11

**Authors:** Kriss D. Pantoja-Pulido, Jonathan Rodríguez, José H. Isaza-Martínez, Margarita Gutiérrez-Cabrera, Ana J. Colmenares-Dulcey, James Montoya-Lerma

**Affiliations:** 1 Departamento de Ciencias Naturales, Exactas y Estadística, Facultad de Ciencias Básicas, Universidad Santiago de Cali, Calle 5 No. 62-00. Cali 760033, Colombia; 2 Departamento de Química, Grupo de Investigación de Productos Naturales y Alimentos, Universidad del Valle, Avenida Pasoancho No. 100-00. Cali 760034, Colombia; jose.isaza@correounivalle.edu.co (J.H.I.-M.); ana.colmenares@correounivalle.edu.co (A.J.C.-D.); 3 Departamento de Biología, Grupo de Ecología de Agroecosistemas y Hábitats Naturales (GEAHNA), Universidad del Valle, Avenida Pasoancho No. 100-00. Cali 760034, Colombia; nathan.rodriguez.g@gmail.com (J.R.); james.montoya@correounivalle.edu.co (J.M.-L.); 4 Laboratório de Entomologia e Fitopatologia (LEF-CCTA), Universidade Estadual do Norte Fluminense, Campos dos Goytacazes 28013, Brazil; 5 Laboratorio de Síntesis Orgánica y Actividad Biológica, Instituto de Química de los Recursos Naturales, Universidad de Talca, Talca 346000, Chile; mgutierrez@utalca.cl

**Keywords:** Mexican sunflower, leaf-cutter ants, insecticidal activity, biological control, cholinesterase activity

## Abstract

Leaf-cutter ants are agricultural and urban pests that defy chemical control methods. Laboratory and field studies have revealed repellent and insecticidal activity by the extracts of *Tithonia diversifolia* (Asteraceae), known as Mexican sunflower, as a promising alternative for the control of the leaf-cutter ant *Atta cephalotes*. This study evaluated the effects of different extracts (non-polar and polar) of *T. diversifolia* dry leaves on worker ants from laboratory colonies of *A. cephalotes* through ingestion and contact. In addition, the biological activity of the extracts as inhibitors of acetylcholinesterase (AChE) and butyrylcholinesterase (BuChE) was evaluated. A dichloromethane extract at 1000 ppm presented the highest insecticidal activity through ingestion, causing 70% and 90% worker ant mortality after five and seven days of treatment, respectively. The acetylcholinesterase inhibition values showed that the dichloromethane presented the best AChE concentration of inhibition (IC_50_) at 73.9 ± 11.06 μg/mL, compared to its fractions, which demonstrates that its activity is potentiated when the crude extract is used. Our results can be attributed to the existence of terpenes and sesquiterpene lactones, which are likely inhibitors of AChE, in *T. diversifolia*.

## 1. Introduction

In Neotropical areas, leaf-cutter ants (Formicidae: Attini) are serious agricultural pests. These ants can be considered the most highly advanced insects in terms of social organization [1]. They maintain a close, ancestral mutualistic relationship with *Leucocoprinus gongylophorus*, a basidiomycetous fungus (Basidiomycete: Lepiotaceae). This fungus is constantly supplied with freshly cut plant material in order to produce the ants’ only food source. In return, the ants disperse the fungus and provide it with a protected environment [2]. In fact, although some synthetic insecticides are used to control and regulate populations of these ants, until now no control agent tested has been fully effective in overcoming the biological and behavioral defenses of these ants under natural conditions [1]. In addition, some insecticides have been banned in several countries due to their high chemical stability and toxicity, which affect and endanger other beings and contaminate the environment [3,4]. Hence, their efficiency is questionable and the development of ecological alternatives to replace these synthetic insecticides is highly desirable.

Through co-evolutionary processes, many plants have evolved a plethora of secondary chemicals (e.g., flavonoids, sterols, coumarins, and sesquiterpene lactones) as defense mechanisms against the herbivore impact of leaf-cutter ants [5]. Some of these compounds exhibit antifungal activity against symbiotic fungi while others act as insecticides, and a few display bio-activity against both ants and symbiotic fungus [5]. In this way, although these secondary metabolites have no immediate or direct application in protecting the plants, an evaluation of their fractions against leaf-cutter ants may produce promising results for their use as control agents, and indicate the route to a commercially viable product.

In the development of control methods for leaf-cutter ants, it is necessary to consider the close, ancestral mutualistic relation that these ants have with their fungus. In fact, their plant preference may be related to the fungus´s nutritional demands in order to survive [6]. Giraldo [7] noted that *Montanoa quadrangularis* trees that were planted and fertilized along with the aster *Tithonia diversifolia* were less frequently attacked by *Atta cephalotes* L., one of the most significant agricultural pests in Latin America. Rodriguez et al. [8] found that, under laboratory conditions, *A. cephalotes* opportunistically forages on *T. diversifolia* leaves when other plant resources are minimal. When colonies are given no alternative but to feed on this plant, the size of the fungus decreases as much as 83.3% [9]. Furthermore, ethanol extract from *T. diversifolia* dry leaves will produce 100% worker ant mortality in only eight days [10]. Recently, in a study conducted under natural conditions, Rodriguez et al. [11] observed that green manure made with *T. diversifolia* will induce the displacement of the fungus-rearing chambers to deeper zones as well as the construction of new chambers outside the areas of manure degradation. Thus, due to its antifungal and insecticidal properties, the sunflower shows great potential for the control of leaf-cutter ants as well as other agricultural pests.

The purpose of this study was to test the insecticidal activity by ingestion and by contact of several fractions of crude *T. diversifolia* extracts, obtained with different solvents, on *A. cephalotes* workers isolated from laboratory colonies. Additionally, the minimum concentration of the extract with the highest toxic effect was identified. Finally, as a complement, taking into account the fact that secondary metabolites such as alkaloids, terpenoids, flavonoids, coumarins and lignans isolated from plants are inhibitors of the acetylcholinesterase enzyme [12], we attempted to evaluate the cholinesterase inhibitory activity of *T. diversifolia* extracts in order to correlate the two types of activities. We hope that these results represent a contribution to the search for novel, environmentally safer molecules for the control of this forest and agricultural pest.

## 2. Materials and Methods

### 2.1. Atta cephalotes Colonies

The colonies were collected in two farms (Entre Quebradas 3°24’58.78” N; 76°35’30.69” W and Corral de Piedra 3°24’51.84” N; 76°35’36.39” W) located at 1350 m above sea level in the rural area of Cali, Colombia, with 80% average relative humidity and 23 °C average temperature. A total of 10 colonies were reared and kept at an average temperature of 24 °C, 75% relative humidity and photoperiod of 12/12 at the Universidad del Valle Biology Experimental Station Laboratory, following protocols described by Valderrama et al. [13]. Workers of medium size (2.6–3.7 mm width of head capsule) were selected for the different bioassays.

### 2.2. Plant Material

*Tithonia diversifolia* plants were grown at Universidad del Valle, Tuluá campus, Colombia. Identity of the species was corroborated by S. Díaz from the Colombian National Herbarium at the Universidad Nacional de Colombia, Bogotá (specimen number COL 552686). *T. diversifolia* leaves were collected and allowed to dry for 10 days at room temperature (25 °C), with 70.4% of the moisture being removed.

### 2.3. Extraction and Fractionation

*Tithonia diversifolia* dry leaves (1144 g) were sequentially extracted by ultrasound with *n*-hexane (three times, 15 min each) followed by 7:3 acetone: water (three times, 15 min each). Both solutions were dried under reduced pressure to obtain the respective extracts: hexanoic (19.13 g, R = 1.7%) and polar (101.10 g, R = 8.8%). The polar extract was sequentially separated through liquid–liquid extraction with dichloromethane and ethyl acetate; thus, generating the dichloromethane extract (10.6 g, R = 7.6%), ethyl acetate extract (2.05 g, R = 1.5%), and aqueous extract (82.31 g, R = 58.8%). Only part of the aqueous extract (50.0 g) was separated with liquid–liquid extraction using water-saturated *n*-butanol to obtain the *n*-BuOH extracts (5.3 g, R = 10.5%) and aqueous residue (42.6 g, R = 85.1%). At each extraction, the solvent was removed through reduced pressure. The dichloromethane extract (7.7 g) was re-dissolved in 7:3 *n*-hexane: ethyl acetate and then fractionated through column chromatography (CC) using silica gel 60 (63–200 µm) Merck™ with successive elutions in 7:3 *n*-hexane: ethyl acetate, 8:2 *n*-hexane: acetone, 1:1 *n*-hexane: acetone, acetone 100%, and methanol 100%. The collected fractions were concentrated under reduced pressure (R = 92.4%). The resulting fractions were monitored via thin layer chromatography (TLC), developed on aluminum silica gel 60 F_254_ (normal and reverse phase RP-18) Merck™ glass plates and read under a UV multiband 254/366 nm lamp. Finally, six fractions were obtained and named Td 2.1.1, Td 2.1.2, Td 2.1.3, Td 2.1.4, Td 2.1.5, and Td 2.1.6.

### 2.4. Toxicity of DMSO and CMC on A. cephalotes Worker Ants

The insecticide activity of the *T. diversifolia* extracts on the *A. cephalotes* worker ants was tested through ingestion and contact bioassays following the methods proposed by Castaño-Quintana et al. [10]. For the ingestion bioassays, the extracts were incorporated into an artificial diet, whereas for the contact bioassays, the extract was applied directly to the mesosoma of the insects using a paintbrush number 6. In the ingestion bioassay, an artificial diet based on glucose and water was supplied to the worker ants [14]. The respective concentrations of each substance to be evaluated were added after the agar was sterilized and cooled. The diet was supplied as cubes every 2 days in Petri dishes. For the contact bioassays, only the artificial diet was supplied, and the substance was applied to the ant once at the start of the experiment. The number of dead ants was tallied daily. This methodology was also used to evaluate the toxicity of dimethyl sulfoxide (DMSO) and carboxymethyl cellulose (CMC), compounds selected to solubilize the different extracts. For the ingestion and contact bioassays, CMC at 0.166% and 0.125%, respectively, were used as blanks. The final concentration of the extracts in each treatment was 1000 ppm. The effectiveness by ingestion of the extract of dichloromethane extracts at 500 and 250 ppm was also compared. Water was used as the control in all bioassays.

### 2.5. Experimental Design and Statistical Analysis

Five experiments with replicate measurement were set up. The experiments with DMSO, CMC, and *T. diversifolia* extracts at 1000 ppm received six distinct independent treatments (Table 1). The ants were selected from five laboratory colonies and distributed randomly in groups of 10 individuals per Petri dish. Ten repetitions were made per treatment in each experiment. The response variable was the number of ants killed per day for 14 days. The experiment in which the concentration of dichloromethane extract was evaluated by ingestion was conducted with four treatments, and the response variable was tallied for the first 10 days.

The statistical analyses of the insecticide activity through contact and ingestion were performed using the software R, version 3.2.2 [15]. Generalized linear models were used in order to determine the effect of the different extracts, blanks (CMC and DMSO) and control through time on ant mortality. The test of multiple contrasts in pairs, based on confidence intervals and using odds ratios (OR) [16], was carried out when a significant effect of the extracts and/or substances was found. This test would indicate the extract most likely to cause mortality in ants. All statistical analyses were performed using a significance level of 5%. All the figures of the experiments were performed using the GraphPad Prism 6.03^®^ software package (GraphPad Software Inc., San Diego, CA, USA).

### 2.6. Cholinesterase Activity

*Tithonia diversifolia* extracts and fractions were subjected to an assay of biological activity as acetylcholinesterase (AChE) and butyrylcholinesterase (BuChE) inhibitors to obtain information on the selective inhibition of both enzymes. Inhibitory activities were evaluated using the methods by Ellman et al. [17]. The samples were dissolved in 96-well plates (50 μL) in phosphate buffer (K_2_HPO_4_ 8 mM, NaH_2_PO_4_ 2.3 mM, NaCl 150 mM, and 0.5% Tween 20, pH 7.6). A solution of AChE/BuChE (50 μL, 0.25 unit/mL) in *Electrophorus electricus* and equine serum, respectively, was added to the same buffer. The assay solutions, except for the substrate, were pre-incubated with the enzymes for 30 min at room temperature. Then, the substrate solution composed of Na_2_HPO_4_ (40 mM), acetylthiocholine/butyrylthiocholine (0.24 mM) and 5,5′-dithio-bis (2-nitrobenzoic acid) (0.2 mM, DTNB, Ellman’s reagent). The absorbance of the reaction at 405 nm was measured for 5 min using a microtiter plate reader (Multiskan EX, Thermo, Vantaa, Finland). AChE/BuChE inhibition was determined for each extract and fraction. The enzymatic activity was calculated as a percentage, compared with the control composed of buffer and enzyme solution. The extracts were tested in dilution intervals ranging from 500 to 15 μg/mL.

Each assay was run in triplicate, and each reaction was repeated three times. The AChE/BuChE IC_50_ values (the concentration that inhibits 50% of AChE/BuChE activity) were determined using regression analysis. The alkaloid galantamine was used as the reference compound. The galantamine IC_50_ was developed in three courses ranging from 250 to 0.122 μg/mL. The values were 0.179 μg/mL (0.486 μM) in the first course, 0.213 μg/mL (0.578 μM) in the second, and 0.193 μg/mL (0.524 μM) in the third, for an average of 0.54 μM.

## 3. Results

### 3.1. Toxicity of DMSO and CMC on A. cephalotes Worker Ants

The deviance analyses generally showed significant differences between at least two of the evaluated substances, although time has a relevant effect on ant mortality for the ingestion and contact experiments. The test of multiple contrasts by pairs showed, for ingestion tests, that 1% and 3% DMSO differ from all others and between themselves. These concentrations induced the greatest ant mortality compared with 0.5% DMSO and with 0.5% and 0.25% CMC. In contact assays, DMSO was the most toxic substance at 0.5%, 1% and 3% concentrations, producing the greatest ant mortality. The two CMC concentrations showed results similar to the control. These results showed that DMSO is too highly toxic to be used as a blank in insecticide activity tests. The least toxic concentration against *A. cephalotes* worker ants was 0.25% CMC. Thus, the solubilization of the *T. diversifolia* extracts with CMC using the lower concentration of 0.25% was preferred for the ingestion and contact bioassays.

Preliminary TLC analysis showed that the composition of some extracts was similar. The chromatographic profile of the ethyl acetate extract was intermediate between the dichloromethane and *n*-butanol extracts. The *n*-butanol extract presented a profile similar to the aqueous residue. Therefore, only the n-hexane, 70% acetone, dichloromethane, and aqueous residue were selected for further evaluation against leaf-cutter ants.

In the ingestion experiment using *T. diversifolia* extracts at 1000 ppm, the deviance analysis showed significant differences between at least two extracts, and additionally, time had a relevant effect on the ant mortality. The multiple paired contrasts showed that the dichloromethane contrast was significantly different from the other treatments, except for the *n*-hexane extract. On the other hand, the *n*-hexane extract was not significantly different from the other extracts or from the blanks and the control. It should be noted that the 70% acetone extract, from which the dichloromethane extract was obtained, did not have the same toxicity. In addition, the aqueous residue behaved like the control, probably due to the high amount of sugars, favoring ant survival. The dichloromethane extract was the most promising as an insecticide, inducing the greatest ant mortality in the shortest time interval: 70% in 5 days and approximately 90% after 7 days.

**Figure 1:**
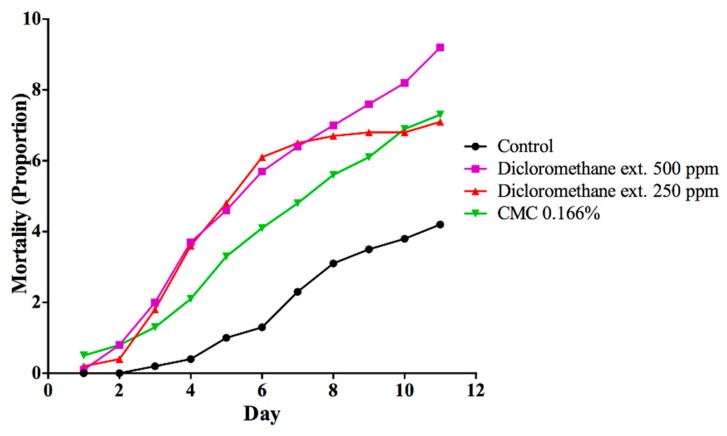
Effectiveness of the *T. diversifolia* dichloromethane extract in different concentrations via ingestion to produce *Atta cephalotes* mortality over time.

**Figure 2:**
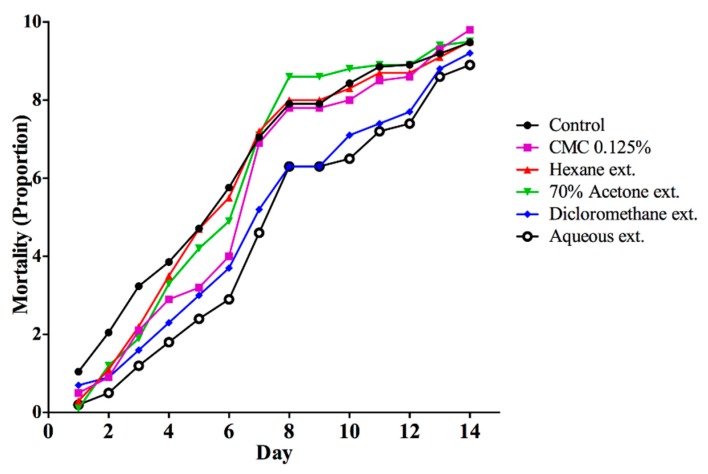
Effectiveness of *Tithonia diversifolia* extracts via contact to induce *Atta cephalotes* mortality over time.

The analysis of the insecticidal activity of the dichloromethane extracts at concentrations below 1000 ppm (i.e., 500 and 250 ppm) showed significant differences between the substances evaluated. Both concentrations caused 70% mortality of worker ants before 9 days, without significant differences. Therefore, the toxicity of dichloromethane extract is perceived as equivalent in both concentrations. Hence, the minimal concentration of dichloromethane for which there was insecticide activity was 500 ppm (Figure 1). In the contact experiment with *T. diversifolia* extracts at 1000 ppm, the mortality was equivalent over the course of the experiment, and no insecticidal tendency was evident. None of the *T. diversifolia* extracts caused ant mortality (Figure 2). The results of this study indicate that the extracts should be reassessed at concentrations higher than 1000 ppm.

### 3.2. Cholinesterase Activity

The results in Table 2 show marked selectivity for acetylcholinesterase (AChE) and no inhibition on butyrylcholinesterase (BuChE) for the different extracts and fractions, although the 70% acetone extract presented the opposite effect, with greater activity over BuChE. All evaluated extracts and fractions inhibited at least 50% of the AChE activity. The dichloromethane and ethyl acetate extracts induced very similar IC_50_ results. The IC_50_ results for the dichloromethane extract fractions are similar and higher than 100 μg/mL. The lowest IC_50_ obtained was 117.72 μg/mL, with the Td 2.1.3 fraction. This demonstrates that AChE inhibitory activity decreased with fractionation.

## 4. Discussion

In natural ecosystems, plant-insect interaction is dynamic, subjected to continual variation and change [18]. In order to reduce insect attack, plants developed different defense mechanisms including chemical and physical barriers [19]. The diversity of plant secondary metabolites has puzzled botanist and natural product chemists for some time. For a long time, the secondary metabolites were considered serve as defense compounds against herbivores. At that time, most botanists did not accept the corresponding defense hypothesis because most of them were not convinced of evolution and adaptive explanations. Botanists preferred the simpler interpretation that secondary metabolites were waste products of primary metabolism and that structural diversity would only reflect a play of nature. Today, adaptive explanations are more favored again to explain the existence and diversity of secondary metabolites [20]. Secondary plant compounds are involved in plant defense against insect herbivores acting as insect repellents, feeding inhibitors, and/or toxins [18]. Terpenoid secondary metabolites occur across a wide range of plant tissue types; those serving as defensive chemicals are often secured in secretory structures. Such specialized structures minimize the risk of auto toxicity but maintain terpene concentrations at sites whose defense is crucial [21].

For all compounds involved with inter-organismal signaling, function can evolve through changes in the recipient organisms rather than through changes in the sending organism [21]. During evolution, plants and insects developed ecological, physiological and biochemical mechanisms to weaken the effect of insect proteinases and plant proteinase inhibitors, respectively [18]. Insect herbivores present complementary adaptations as a response to each defensive adaptation in host plants. Plant chemical composition is variable and represents a challenge for insect feeding. However, insects possess a powerful assemblage of enzymes that constitute their defense against chemical toxicants [22].

Secondary metabolites such as sesquiterpene lactones, diterpenes, flavonoids and sterols [23,24,25] and other classes of compounds, such as phytosterols, xanthans, coumarins, ceramides, chromones, and chromenes [26,27] have been isolated from *T. diversifolia* extracts. Several biological studies have shown that these compounds have, among others, bio-insecticidal, and repellent activity [24] mostly due to the sesquiterpene lactones, which are present in large quantities in Asteraceae.

Low-polarity compounds such as alkanes, fatty alcohols, other linear hydrocarbons, triterpenols, sterols, and carotenoids were likely acquired with the hexane extraction, whereas fatty acids, esters, sesquiterpene-type terpenes, terpenoids, and phenylpropanoids, among others, were extracted with the 7:3 acetone: water mixture. In contrast, extraction with dichloromethane, while acquiring compound residues similar to those of the *n*-hexane extract, are mostly of medium polarity and were pulled during the extraction with 70% acetone. The lipid residual entrained by dichloromethane probably influences how the phenolic and sesquiterpenic secondary metabolites are perceived by the insects. Hence, when they are isolated and concentrated in the *n*-hexane extract, their activity is not intensified. The compounds that contain the lipid fractions of some plant extracts have showed repellent activity against *A. cephalotes* leaf-cutter ant; many of these compounds are terpenoids [28]. Other studies have shown that extracts of epicuticular lipids have allelochemical effects in terms of phago-inhibition or toxicity. These components have been associated with the resistance of plants to herbivores [29]. On the other hand, the diets that contained dichloromethane extracts were not ingested or crumbled when supplied to the ants. This behavior was not observed in other treatments. Another unusual behavior noted was ant ‘numbness’, evidenced by the lack of movement and inactivity in the Petri dishes. These observations lead us to suspect that the insecticidal activity of the *T. diversifolia* dichloromethane extract might induce a repellent effect in the ants. These behavioral changes intensified as the experiment progressed.

There are some reports in the literature regarding chloroform extracts of other Asters against leaf-cutter ants. Terrance and Wiemer [30] demonstrated such activity with *Melampodium divaricatum* leaf and branch extracts against *A. cephalotes* ants in laboratory colonies. Similarly, Okunade and Wiemer [31] found that *Eupatorium quadrangulare* leaf extract, rich in sesquiterpene lactones, had a repellent effect against this ant species. On the other hand, Ambrósio et al. [23] provided evidence for anti-feeding or deterrent activity for dichloromethane *T. diversifolia* leaf extract, showing that lepidopteran larvae of *Chlosyne lacinia* avoid sesquiterpene lactones of glandular trichomes. Fourteen sesquiterpene lactones, one flavonoid, and one diterpenoid were isolated in that work. Aqueous and ethanolic extracts of this plant also showed insecticidal activity on *Macrotermes bellicosus* (Termitidae) [32]. Adedire and Akinneye [33] found that ethanolic extracts of *T. diversifolia* adversely affect oviposition, adult emergence and survival of *Callosobruchus maculatus* (Chrysomelidae). Oyewole et al. [34] found that *T. diversifolia* essential oils have repellent action against the mosquito *Anopheles gambiae* (Culicidae) due to the volatiles present in the plant. In this regard, Dai et al. [35] reported on the main constituents of the essential oil obtained through hydro-distillation; the oil composition included an abundance of monoterpenes (52%), sesquiterpene hydrocarbons (22.7%), and oxygenated sesquiterpenes (11.8%).

The *T. diversifolia* insecticide activity obtained with the dichloromethane extraction via ingestion on worker ants isolated from laboratory colonies could be attributed to the presence of sesquiterpene lactones and other previously mentioned terpenic compounds, which may be responsible for the repellent and insecticide activity. That is, these compounds are the usually powerful chemical weapons that plants such as *T. diversifolia* produce to defend themselves against herbivores. These phytotoxins can be released from the plants by volatilization, root exudation, leaching, and decomposition of the plant material [36]. Therefore, phytochemical separation of this extract is required for conclusive identification of the active substances. However, components that occur in a lower abundance also contribute to biological activity, reflecting the importance of the compositional complexity to confer bioactivity [37]. This is the case of complex mixtures of terpenes, which may also be present in dichloromethane extract. It has been hypothesized that a synergistic phenomenon between the secondary metabolites might result in higher bioactivity compared to the isolated compounds [38]. This occurs because plants generally present defenses as a set of compounds rather than as individual compounds [37]. Thus, the results of the ingestion experiment allow us to order the extracts according to their insecticidal activity at 1000 ppm, (i) high activity: dichloromethane extract; (ii) low activity: hexane extract, and (iii) no activity: 70% acetone extract and aqueous residue.

The transmission of nerve impulses is regulated by the AChE enzyme, which is present in nervous tissue of both humans and insects. The inhibition of this enzyme causes the accumulation of acetylcholine in the synapses and, consequently, the interruption of the regular transfer of nerve impulses, evidenced by muscular convulsions and paralysis, as well as other characteristics of self-poisoning by excess acetylcholine [39,40].

Although both glycoproteins (AChE and BuChE) hydrolyze choline esters in nerve synapses, the main difference lies in their composition and the size of the active site. Amino acid residues are responsible for AChE and BuChE inhibitory specificity. Different compounds can attach to any of the sub-sites of the active centers of AChE or BuChE and inhibit them selectively, such as BW284C51 for the AChE anionic sub-site and iso-OMPA, ethoxypropazine, and bambuterol for BuChE [41]. However, the use of some synthetic compounds has led to the appearance of resistance in some insects, as a result of modifications to AChE [42,43]. According to reports, plant metabolites, such as terpenoids, exhibit anticholinesterase action [40,44]. In response to this problem, some research has focused on the use of natural products with insecticidal activity. Compounds such as alkaloids (isoquinolines, indolics, piperidines, and triterpenoids); terpenes such as monoterpenes, sesquiterpenes and derivatives; diterpenes; triterpenes; steroidal lactones; flavonoids and derivatives; coumarins; stilbenoids; phenylpropanoids and phenolic compounds have shown strong AChE inhibitory activity [42,45,46].

From our results, dual inhibition can be inferred to have been generated only by the raw 70% acetone extract, and with the liquid-liquid fractionation, the inhibition becomes specific for AChE due to the metabolites in the different extracts of *T. diversifolia*. This hypothesis is based on the values of IC_50_ > 500 μg/mL for BuChE, which demonstrates that no significant enzyme inhibition was achieved, probably because the compounds do not bind to BuChE active sites. The highest inhibition of the AChE enzyme was obtained with dichloromethane and ethyl acetate *T. diversifolia* extracts, which presented the lowest and closest IC_50_ values compared to all other extracts, 73.92 and 65.63 μg/mL, respectively. Therefore, the isolation and characterization of the secondary metabolites to evaluate the AChE activity in purified fractions and to establish differences between the measurements of their respective extracts would be interesting. The AChE inhibitory activity obtained with the dichloromethane fractions demonstrates that the inhibition decreased upon fractionation, indicating that the activity is potentiated when the crude extract is used.

## 5. Conclusions

Our laboratory studies have revealed insecticidal activity by the extracts of *T. diversifolia*, known as Mexican sunflower, as a promising alternative for the control of the leaf-cutter ant *A. cephalotes*. The dichloromethane extract of *T. diversifolia* leaves at 1000 ppm presents the greatest insecticidal activity, causing 70% mortality of *A. cephalotes* worker ants five days after ingestion. In addition, the values of acetylcholinesterase activity and the AChE IC_50_ for ethyl acetate and dichloromethane extracts (65.63 and 73.92 μg/mL, respectively) were lower than the dichloromethane fractions, demonstrating that the acetylcholinesterase is more satisfactory when crude extracts, rather than purified fractions, are used. Our results confirm that the insecticidal activity of *T. diversifolia* on *A. cephalotes* is due to secondary metabolites that inhibit the acetylcholinesterase enzymatic activity; hence, explaining the lack of movement and inactivity by the ants observed in the Petri dish essays. Because other reports have found an association between the toxic activity of *T. diversifolia* due to its terpenes and sesquiterpene lactones, future work should follow up with the isolation and identification of the major metabolites that generate insecticide, repellent and acetylcholinesterase activity against *A. cephalotes.*

## Figures and Tables

**Table 1:** Experimental design used in the bioassays with *Atta cephalotes.*

Treatment	DMSO and CMC		*T. diversifolia* Extracts at 1000 ppm		Comparison Dichloromethane Extracts
	Ingestion	Contact	Ingestion	Contact	Ingestion
**T1**	Diet	Diet	Diet	Diet	Diet
**T2**	Diet + CMC 0.25%	CMC 0.125%	Diet + C1	C2	Diet + C1
**T3**	Diet + CMC 0.5%	CMC 0.25%	Diet + Hexane extract in C1	Hexane extract in C2	Diet + Dichloromethane extract (250 ppm) in C1
**T4**	Diet + DMSO 0.5%	DMSO 0.5%	Diet + Acetone 70% extract in C1	Acetone 70% extract in C2	Diet + Dichloromethane (500 ppm) in C1
**T5**	Diet + DMSO 1%	DMSO 1%	Diet + Dichloromethane extract in C1	Dichloromethane extract in C2	−
**T6**	Diet	Diet	Diet	Diet	Diet

C1: CMC at 0.1666% concentration, C2: CMC at 0.125% concentration, DMSO: dimethyl sulfoxide, CMC: carboxymethyl cellulose.

**Table 2:** Cholinesterase (acetylcholinesterase (AChE) and butyrylcholinesterase (BuChE))-inhibiting activities of *Tithonia diversifolia* extracts and fractions.

Extract/Fraction	Weight (mg)	AChE IC_50_ (μg/mL)	BuChE IC_50_ (μg/mL)
Hexane extract	19.13 × 10^3^	260.57 ± 0.001	>500 *
70% Acetone extract	101.10 × 10^3^	109.2 ± 12.18	60.6 ± 12.17
Dichloromethane extract	10.60 × 10^3^	73.9 ± 11.06	>500 *
Ethyl acetate extract	2.05 × 10^3^	65.6 ± 9.06	>500 *
Butanol extract	5.25 × 10^3^	105.0 ± 18.13	>500 *
Aqueous residue extract	42.57 × 10^3^	130.5 ± 12.23	>500 *
Td 2.1.1 fraction	45.1	>500 *	>500 *
Td 2.1.2 fraction	936.1	144.5 ± 0.02	>500 *
Td 2.1.3 fraction	784.5	117.72 ± 0.005	314.6 ± 0.009
Td 2.1.4 fraction	2783.5	155.64 ± 0.005	>500 *
Td 2.1.5 fraction	776.4	186.77 ± 0.003	>500 *
Td 2.1.6 fraction	1893.7	119.0 ± 0.02	>500 *
Galantamine		0.54 μM ± 0.7	8.80 μM ± 0.5

* Values not determined, AChE/BuChE IC_50_ > 500 μg/mL.
